# ‘People need to be a bit more understanding that my body is wrecked’: A qualitative exploration of inpatient hospital care for people living with multiple long-term conditions

**DOI:** 10.1186/s12916-026-05181-4

**Published:** 2026-10-16

**Authors:** Sue Bellass, Thomas Scharf, Miles D Witham, Lynsey Threlfall, Chris Plummer, Avan A Sayer, Rachel Cooper

**Affiliations:** 1 AGE Research Group, Translational and Clinical Research Institute, Faculty of Medical Sciences, Newcastle University, Newcastle upon Tyne, UK; 2 NIHR Newcastle Biomedical Research Centre, The Newcastle upon Tyne Hospitals NHS Foundation Trust, Cumbria, Northumberland, Tyne and Wear NHS Foundation Trust and Newcastle University, Newcastle upon Tyne, UK; 3 Population Health Sciences Institute, Faculty of Medical Sciences, Newcastle University, Newcastle upon Tyne, UK; 4 Digital Services, The Newcastle upon Tyne Hospitals NHS Foundation Trust, Newcastle upon Tyne, UK

**Keywords:** Multiple long-term conditions, Multimorbidity, Hospital care, Qualitative research

## Abstract

**Background:**

Living with multiple long-term conditions (MLTC) is becoming increasingly common with far-reaching consequences for individuals and healthcare systems. People with MLTC often face complex care pathways through health systems - especially hospitals, which are largely configured for specialist treatment of single conditions - yet evidence on people with MLTC’s lived experience in the hospital setting is limited. This study aimed to understand the hospital care experiences of people living with MLTC who had recently had an inpatient stay.

**Methods:**

People with MLTC who had experienced an inpatient stay in hospital within the previous six months were recruited via three hospitals in England and via patient networks. Semi-structured one-to-one interviews were conducted with each participant, focussing on their experiences of care from admission to discharge. An inductive thematic analysis was undertaken.

**Results:**

A total of 44 people (mean age 68.4 years, 23 women) who reported living with between 2 and 11 long-term conditions, the majority of whom (96%) reported that their most recent hospital stay was unplanned, participated in the study. Three themes were constructed from the interview data, reflecting perceptions at individual, interpersonal and organisational levels. Participants’ experiences were shaped by *internalised narratives of hospital care*, where care was expected to be focussed primarily on single conditions within a resource-constrained environment. Relationally, the degree of *alignment between clinician and patient knowledge* on conditions was a key contributor to whether hospital care was experienced positively or negatively, and participants’ perceptions of *organisational constraints to holistic care* gave insights into their views on system-level barriers shaping the provision of care for MLTC in the hospital setting.

**Conclusions:**

Experiences of inpatient hospital care for people with MLTC are complex, diverse and shaped by expectations of care in a specialist setting configured to provide care for single conditions. Healthcare professionals should incorporate patients’ experiential expertise into decision-making processes through consultation with people with lived experience of MLTC. Redesigning hospital services to provide holistic care will require flexibility to respond to the wide spectrum of MLTC experiences.

**Supplementary Information:**

The online version contains supplementary material available at https://doi.org/10.1186/s12916-026-05181-4.

## Background

Multiple long-term conditions (MLTC), defined as the coexistence of two or more long-term health conditions [1], have far-reaching consequences for both individuals and healthcare systems [2, 3]. As the concept of MLTC (or multimorbidity, as it is also referred to) considers all co-existing conditions equally, it is distinct from comorbidity which focuses on an index condition and considers the presence of additional long term conditions in relation to the specified one [4, 5]. MLTC have been identified as a global health priority [1], since the prevalence in all world regions and settings is high [6, 7, 8], and projected to continue rising [9, 10]. This is exemplified by recent evidence showing an increase in prevalence of MLTC among people admitted to hospital in the North East of England from 50% in 2018–2019 to 61% in 2021-22 [7].

Living with MLTC has myriad adverse effects for individuals and their families related to high symptom and treatment burdens [11], leading to poorer physical function [12] and other adverse outcomes. In addition, evidence that people living with MLTC face challenges navigating fragmented health and social care services is accumulating [13, 14, 15]. People living with MLTC are disadvantaged by healthcare systems configured to provide specialist care for single-organ conditions such as inpatient hospital settings [16], exemplified by their higher rates of inpatient mortality and readmission to hospital [17, 18], longer stays [19], and lower levels of satisfaction with hospital care [20].

Developing a clearer understanding of people living with MLTC’s experiences of inpatient hospital care is required to inform the transformation of hospital services. Without this it will be difficult to effectively meet the needs of the growing proportion of the population living with MLTC. Recent scoping reviews have summarised the existing knowledge base on perceptions of MLTC hospital care, highlighting the importance of person-centred care and service integration [21, 22]. However, whilst studies have highlighted experiences of hospital care, limited attention has been paid to how experiences are shaped by people’s expectations of care. Further, much of the evidence is from research on comorbid conditions – where another condition (or conditions) occurs alongside and often compounds the effects of an index condition – rather than MLTC, in which no single diagnosis takes primacy [23]. In the United Kingdom (UK), qualitative studies of perceptions of hospital care in the context of MLTC have been identified [24, 25], but these studies focus on specific sub-groups (people in the last year of life and pregnant women).

While offering important insights, the existing evidence base therefore has limitations. The heterogeneity of MLTC, and the high prevalence of MLTC among people admitted to hospital, require an understanding of the challenges that MLTC present that go beyond specific comorbidities, for which care pathways may already be established. In this study, nested within a wider research collaborative investigating hospital care and MLTC [26], we aimed to understand how people living with MLTC experience inpatient hospital care within the National Health Service (NHS) in England, to explore aspects of care that are appreciated by people with MLTC, and to identify priorities for service redesign.

## Methods

A qualitative semi-structured interview study was conducted with people living with MLTC who had experienced at least one inpatient stay in an NHS hospital within the preceding six months. NHS Health Research Authority ethical approval for the study was granted by Wales Research Ethics Committee 3 [reference no. 23/WA/0045].

### Study preparation

The ADMISSION Research Collaborative benefitted from regular advice and guidance from a diverse panel of people living with MLTC and carers (ADMISSION Patient Advisory Group; PAG) from pre-award to the end of the funded period. At the pre-award stage, the PAG highlighted the need for a qualitative study to capture the lived experience of MLTC hospital care [26]. Post-award, discussions and an online survey with the PAG informed the topic guide and study design. In addition, members of the PAG reviewed public-facing documentation and took part in pilot interviews to support refinement of the interview topic guide.

### Sampling and recruitment

Given the highly heterogeneous nature of MLTC, purposive sampling was undertaken to construct a diverse sample of people based on a range of characteristics including age, gender, ethnicity and condition combination. Identification of potential participants was undertaken by hospital clinicians at three NHS sites in England, two in the North East (The Newcastle upon Tyne Hospitals NHS Foundation Trust; Gateshead Health NHS Foundation Trust) and one in the North West (Northern Care Alliance NHS Foundation Trust). Clinicians supporting study recruitment approached potentially eligible patients nearing discharge to request consent for contact by the research team, providing the potential participant with a study information sheet. Recruitment was supplemented by posters in waiting areas at NHS sites, promoting the study via VOICE, a network of public contributors hosted by Newcastle University, and through advertisement in Healthwatch Leeds. The study was also promoted on X (then Twitter) and through personal networks. Several voluntary sector organisations serving groups of people likely to be living with MLTC in the North East and North West of England were also contacted with a request to promote the study across their networks.

Inclusion criteria for the study were: people aged 18 years or older with capacity to consent who were living with any combination of two or more long-term conditions (defined as mental, physical or long-term infectious conditions that had lasted, or were expected to last, for more than a year), and who had experienced at least one inpatient stay of at least one night in an NHS hospital within the last six months. Data collection took place between November 2023 and December 2024. The sample was closely monitored by the research team throughout the recruitment process to identify and address under-represented characteristics. Regular meetings were held with clinicians at recruiting NHS hospital sites to provide updates on the sample and advise of target characteristics.

### Arranging and conducting interviews

Potential participants who had consented to contact by the research team were approached according to their preferred time and method of contact (either telephone or email), which they had been asked to provide on the consent to contact form. The researcher (SB), an experienced post-doctoral qualitative researcher, made three attempts to contact each potential participant.

If the potential participant was eligible and willing to take part in the study, the researcher arranged a mutually convenient time for interview, either in-person, via telephone or videoconferencing software to enable the participant to choose their preferred means of engagement. Participants could choose to be accompanied by a friend or relative if they wished, although only the participants’ data would be included in analyses. Prior to the interview taking place, consent was given by the participant either via a signed paper consent form or audio recording. An interview topic guide on hospital care, shaped by extant literature and developed through consultation with the research team and the ADMISSION Patient Advisory Group (PAG), was employed flexibly (see Additional File 1). Sociodemographic and self-reported health condition data were collected during the interview. No incentives to participate were provided.

All interviews were audio-recorded and transcribed in intelligent verbatim style (excluding filler sounds such as ‘um’ and ‘ah’) by a reputable transcription company with extensive experience of transcribing research interviews. Transcripts were checked and anonymised before being imported into NVivo 14 for coding. All data were stored behind an institutional firewall in folders with access limited to study team members.

### Analysis

Data were analysed inductively using thematic analysis [27, 28]. Following familiarisation with the data, achieved through thorough reading of the transcripts, the researcher generated preliminary codes from five transcripts to develop an initial coding frame, which was then iteratively refined through applying the coding structure to a further five transcripts. This structure was then applied to the data, revising where necessary to capture additional patterns and meanings. Themes and sub-themes were developed through iterative processes which were sense-checked with other authors through discussion at team meetings. The theme structure was then further refined to ensure representation of the dataset.

### Reflexivity statement

The research was conducted by the lead author (SB), an experienced qualitative health researcher with a background in social science, with supervision and guidance from the authorship team whose members have expertise in geriatric medicine, acute medicine, cardiology, epidemiology and social gerontology.

The lead author’s disciplinary background privileges understanding the agency of the study participants as patients acting within the institution of a healthcare system with established norms, cultural frameworks and temporal patterns. As a non-clinician influenced by interactionist traditions, and with a theoretical grounding in the sociology of chronic illness, the lead author’s interests in relationality, temporality and embodied experiences of illness shaped the analysis. To address the limitations of this approach, regular discussions were held with other members of the authorship team to enable the lead author to view the analysis through other disciplinary lenses, enabling continuous critical reflection during theme construction.

Throughout the data collection and analytic processes, the lead author had an informal caring role for a family member who lived with MLTC and had frequent inpatient NHS hospital stays. Although not disclosed to participants, this lived experience may nonetheless have shaped the distance in the researcher-participant relationship, facilitating a process where the lead author may have understood participants’ experiences in relation to those of her family member. Remaining conscious of this potential influence and maintaining an ongoing dialogue with study supervisors as the collection and analysis proceeded, supported continual reflexive engagement with the data.

## Results

**Fig. 1:**
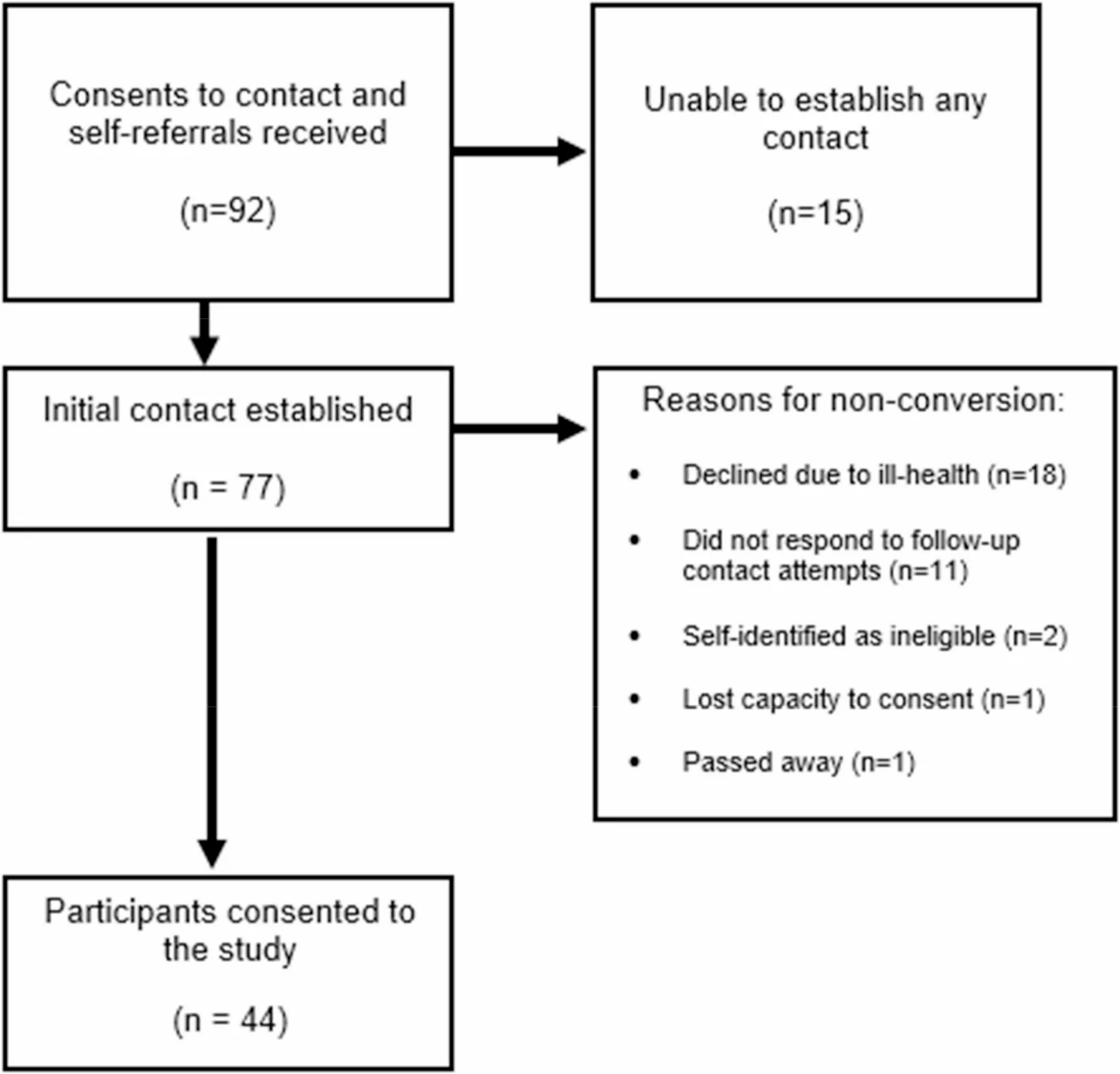
Participant conversion flow diagram

Of a total of 92 consent to contact forms or self-referrals received by the research team, 44 participants were consented to the study (Fig. 1). Semi-structured interviews were conducted with this diverse sample of 44 adults (23 women) aged 34–87 years old (mean 68.4) who had all experienced an inpatient stay in an NHS hospital within the preceding six months. Twenty-three participants lived in the North East of England, 20 in the North West and one in Yorkshire. Most participants (38/44, 86%) were recruited from NHS sites, with an additional four from VOICE, one via an advertisement in Healthwatch Leeds and one from personal networks. We did not recruit any participants via X or any other social media platform and, perhaps because there were no incentives to join the study, we did not experience inauthentic participation, where people falsify identities or experiences to participate in research studies [29].

Most interviews (29/44, 66%) were conducted in-person in participants’ homes, with 13 (30%) taking place via telephone, and two (4.5%) via videoconferencing software. Interviews lasted between 21 and 129 min.

Participants reported living with between two and 11 long-term conditions. With the support of a clinician, the conditions were grouped according to affected body systems (Additional file 2) using a categorisation system developed by members of the research team (MDW, AAS, RC) based on ICD-10 codes [30]. Most participants (42/44, 96%) had experienced an unplanned admission during their most recent hospital stay and had stayed in hospital for less than a week (38/44, 86%) (Table 1). Admission was precipitated for various reasons including abnormal potassium levels, respiratory difficulties and septicaemia. The sample was diverse according to a range of different sociodemographic characteristics (Table 1 and Additional file 3).

**Table 1:** Characteristics of study participants

Characteristic	Frequency *N* (%)
**Gender**	
Female	23 (52)
Male	21 (48)
**Age (y)**	
30–39	3 (7)
40–49	2 (5)
50–59	4 (9)
60–69	8 (18)
70–79	18 (41)
80–89	9 (21)
**Index of Multiple deprivation**	
Most deprived decile	5 (11)
2nd	11 (25)
3rd	5 (11)
4th	5 (11)
5th	1 (2)
6th	3 (7)
7th	3 (7)
8th	2 (5)
9th	4 (9)
Least deprived decile	4 (9)
No code	1 (2)
**Ethnicity**	
White British	43 (98)
White African	1 (2)
**No. of self-reported long-term conditions**	
2–3	14 (32)
4–5	18 (41)
6–7	11 (25)
≥ 8	1 (2)
**Body system/s (based on ICD-10 chapters) for long-term conditions reported**	
Cancer	11 (25)
Cardiovascular disease	19 (43)
Digestive disease	8 (18)
Ear disease	5 (11)
Eye disease	3 (7)
Haematological disorder	2 (5)
Infectious disease	1 (2)
Mental and behavioural disorder	3 (7)
Metabolic and endocrine disorder	13 (30)
Musculoskeletal disease	19 (43)
Neurological disease	6 (14)
Respiratory disease	17 (39)
Urogenital disorder	7 (16)
**Type of hospital stay**	
Unplanned	42 (96)
Planned	2 (5)
**Length of most recent hospital stay**	
Overnight	6 (14)
2–4 nights	26 (59)
5–6 nights	6 (14)
1–4 weeks	3 (7)
More than 4 weeks	3 (7)
**Inpatient stays in last year**	
1	22 (50)
2–4	17 (39)
≥ 5	5 (11)

Three interrelated yet distinct overarching themes with sub-themes were developed (Table 2), which reflected perceptions at individual, interpersonal and organisational levels: internalised narratives of NHS hospital care, perceptions of alignment between clinician and patient knowledge, and organisational constraints to holistic care.

**Table 2:** Theme structure

Themes	Sub-themes
**Internalised narratives of hospital care**	Hospital as inherently specialised
	Hospital as a resource-constrained environment
	Transparent routines, opaque processes
**Alignment between clinician and patient knowledge**	Involvement in the care process
	Medication management
**Organisational constraints to holistic care**	Integration of services
	Information continuity
	Perceptions of clinical training and guidelines

1. **Internalised narratives of NHS hospital care**

Evident throughout the accounts of study participants were the ways in which public narratives of hospital care shaped expectations of the care they would receive. This theme comprised three sub-themes including hospital as inherently specialised, hospital as a resource-constrained environment, and transparent routines but opaque processes.

- Hospital as inherently specialised

Study participants generally understood hospitals to be specialist, short-stay environments, where acute problems would be resolved and they could return to their prior life on discharge. Apart from expecting to receive medication for their long-term conditions, or an acknowledgement of limitations caused by other conditions, participants generally did not anticipate that care would be provided for conditions that had not precipitated their hospital admission. As one participant explained:

“They’ll monitor you if your heart is not working properly because they’ve got the machines, you know what I mean, but you’re specifically just for whatever you go in for. You can’t go in and say, “Can you check my back and my arm?” (GHM01; man, 69, eleven long-term conditions (LTC)).

Other participants expressed similar views, with one man noting that he had not expected to receive care for another of his health conditions: “It’s a totally separate issue. In other words, we specialise in this, we specialise in that, and that’s all we do” (NCM07, man, 84, three LTC). Mirroring the single-condition focus of hospitals, participants frequently conceptualised their health biomedically, adopting language relating to body systems, diagnostic labels or specialisms when referring to their inpatient stays, ‘I’ve been back in again with fluid on my lungs…they drained four litres…It’s the heart and kidneys. My kidneys have gone down to 20%’ (NTM07, man, 51, six LTC) rather than symptoms resulting from LTC.

b) Hospital as a resource-constrained environment

Prominent in the accounts were descriptions of hospital as a resource-constrained environment due to a combination of insufficient staffing for the high demand for care. Media reports or personal experience of long waits on hospital corridors in emergency departments were commonly referred to, with participants perceiving beds to be the currency of the hospital care system. This could result in participants questioning the legitimacy and deservingness of their bed occupation based on an assessment of their symptoms. One participant noted, for instance:

“I felt like a fraud, because I didn’t actually feel ill. I just felt very tired and I had a very dry mouth. Those are the only symptoms I had, was my problem. I kept thinking, “Oh, there are all these people coming in that need the bed and I’m sat here just…” (NCF02, woman, 72, four LTC).

Living with MLTC could be a complicating factor in the duration of bed occupancy, and the desire to relinquish it. Discharge processes could be delayed, for instance, due to waiting for advice or information from another specialism, leading to participants feeling frustrated by being unable to surrender their bed for someone more unwell. One participant, for instance, noted:

“They said, ‘We’re still waiting for renal,’ and so I just waited and then one of the junior doctors came and I said… I wasn’t rude, I wasn’t rude or anything like but I just said to her, ‘I’m just bed-blocking.’ I said, ‘If renal don’t come down in the next couple of hours, then I’m sorry but I’m going home.’” (NCF01, woman, 83, six LTC).

The awareness of resource limitations in the hospital setting could impact not only perceptions of legitimate occupation of a bed but also receipt of nursing care. Participants frequently commented on the busyness of staff, particularly nursing staff, who were often sympathetically described as being “rushed off their feet” (NCF06, woman, 75, six LTC). Some participants felt that this affected the ability to provide person-centred care, with one man noting ‘they’re always too busy for an individual person. “I think, ‘Oh, they don’t care about me’” (NCM03, man, 80, four LTC), but generally an awareness of the pressures affecting the hospital system shaped the level of care they expected to receive. For example, one man noted:

“Because, you know, if you’ve got another 200 people to look after, they’re not going to get round to you as often as if there were 50 people… they’re not going to come round and, you know, mop my fevered brow or anything, are they? They’ve got a job to do” (NNM02, man, 72, five LTC).

The sense of busyness could result in a perception that staff did not have capacity to respond to health conditions other than the reason they had been admitted to hospital. Some participants perceived that, due to workload pressure, staff would only respond to conditions if they became problematic during a hospital stay. One participant stated:

“They don’t have time to tie everything together when you’ve got multiple illnesses. It’s just kind of like, “Well we’ll do this and, if anything happens with your other problems then, we’ll deal with that.” (NCM10, man, 48, four LTC).

c) Transparent routines, opaque processes

The third element of this theme contrasts participants’ expectations and experiences of hospital routines with their pathways through the hospital system. Participants frequently described routinised aspects of their time in hospital – monitoring of temperature and blood pressure, the arrival of food and medication, visiting times and night-times – as anticipated and predictable events. However, such routines contrasted with participants not knowing or expecting to know how their journey through the hospital would unfold from admission to discharge. Participants typically expected long waits on hospital corridors, occasionally referring to “horror stories” (NCF09, woman, 62, three LTC) of such occurrences in the media. Whether they would be admitted to a ward, or remain in an assessment unit, was largely unknown. Although several participants described being asked by staff if they would like to delay discharge for an additional day, in general participants neither knew nor expected to know the timing or stages of their pathway through the hospital system, instead often expressing surprise when processes involving receiving care from more than one department ran smoothly or more quickly than expected:

“I was in hospital, the following day, having this little op so the transfer from that one department to this went as smooth as anything…That was all within two days…That was a big shock to my system.” (NCF07, woman, 86, five LTC).

2. **Alignment between clinician and patient knowledge**

Study participants gave both positive and negative accounts of their hospital care, with a key difference between the accounts being whether patients perceived their and the clinicians’ knowledge of their conditions to align. Participants giving a positive narrative generally felt involved in the care process, confident that their medications were well-managed, trusted their clinicians and were able to use their agency to shape decisions about their care. In contrast, people giving negative accounts felt excluded from care decisions, reported being unable to influence the care process, felt their medications were not managed well and expressed doubts in clinicians’ knowledge and skills.

- Involvement in the care process

Key to feeling involved in the care process was shared decision-making during the hospital stay. Participants who described discussing and agreeing treatment options with medical staff held positive views of their care. One man, who has had long-term relationships with clinicians through developing a kidney disorder as a child, noted:

“Every doctor I speak to, we have a two-way conversation. They listen to what I’m going through. They give me their feedback. And that’s brilliant, and it has worked well.” (NCM04, man, 37, three LTC).

In contrast, participants reported frustration if they felt that the clinicians were not taking into consideration their other LTC. One participant, for instance, noted that “all the time I was in there I was concerned about my tummy, but nobody else was” (NNF03, woman, 76, four LTC). Another stated “you’re constantly having to explain that you’ve got sleep apnoea, that you’ve got breathing issues, that you’ve got Crohn’s, that you’ve got problems with your kidneys. Then the next person comes in and you have to go through it all [again].” (NCM10, man, 48, four LTC).

There were several instances of participants providing information relating to other conditions that they felt were not taken into account by hospital clinicians. One woman, aged 39, attending for an operation, described a situation in which her concerns about incontinence were not responded to, causing frustration and embarrassment:

“I told people at my pre-assessment…’I have these three conditions. I have Crohn’s disease, I have - ‘I remember telling, at my pre-assessment, that, ‘I struggle with bowel incontinence. So, obviously, most of the time, it’s controlled, but I can’t tell you what all the medication you use in putting me under for surgery is going to do to my poor bowels.’ So, I said, ‘Do you need me to - do you provide incontinence pads at hospital? Should I be wearing one? Should I bring my own in?’ And I remember, when I got to hospital, and I woke up from my surgery, that hadn’t been accounted for.” (NNF02, woman, 39, three LTC).

Similarly, frustrations could occur when there was a sense that staff would treat all patients who had gone through a particular procedure in the same way, overlooking other LTC that the person lived with. For example, one participant who had had a kidney transplant, noted:

“Some of them [nurses] were just coming in and being like, ‘You need to get in your chair, you need to get up.’ And I think, when you’ve had your stomach cut open and your body is already weak from everything else you’ve been through, it’s not as easy as someone that has never been through anything before… Whereas when I was there, sometimes I just needed to rest. But I just feel like, with my care, people need to be a bit more understanding that my body is wrecked, and sometimes I might not bounce back as quickly as someone else.” (NTF06, woman, 40, four LTC).

b) Medication management

Care for other LTC in the hospital setting was more frequently achieved through medication management rather than through contact with other specialist teams in the hospital setting. Varying experiences with medication management were reported by study participants. For some, medication for all of their conditions was received in a timely way:

“I just said, ‘This is what I’m on,’ and they said, ‘Oh, that’s fine, so everything’s okay.’ So, the next thing, I woke up in the morning and, ‘Here are your tablets,’ and, ‘Oh, right, thank you.’” (NCF02, woman, 72, four LTC).

For other participants, receiving their usual medication for LTC was more problematic. Some reported not receiving the right medication, or receiving it at the wrong time of day, leading them to use medication they had taken into hospital, or to ask family members to bring medication from home when visiting. One person noted:

“I took them [tablets] in with me because I know I take an awful lot of tablets. They said, ‘Well…’ …I said, ‘What are you giving me them for? I’ve got my own.’ ‘Well we’ve done these now.’ I said, ‘Well you’ve not given me enough.’ I said, ‘I take… Baclofen three times a day’, they were only giving me one. I went, ‘No, I have three so I’m going to take my own.’ Things like that, it’s just… They’re not reading the notes properly, are they?” (NCF03, woman, 63, four LTC).

Other participants reported similar tensions with staff when self-medicating in the hospital setting. One participant, for instance, living with severe chronic obstructive pulmonary disease (COPD) and emphysema amongst other conditions, reported that the staff had not read her records, and that her nebulisers had not been provided:

“Mistakes were made and I didn’t get all my meds. I mean I was supposed to have my nebs [nebulisers] four times a day. I didn’t get any… Well, fortunately, I have my own portable one… They don’t like using your own equipment. They want you to use theirs, but if they don’t bring it to you, you can’t use it.” (NCF04, woman, 71, six LTC).

3. **Organisational constraints to holistic care**

Several participants described their perceptions of organisational constraints which impacted holistic, person-centred care in the hospital setting. This theme comprises two sub-themes: integration of services, and perceptions of clinical training and guidelines.

- Integration of services

Some participants described receiving integrated care during an inpatient stay, but the effectiveness of this depended on how well the specialties communicated with each other. For instance, one participant described how his kidney transplant depended on integration between renal and neurology specialists:

“When I was on dialysis I had to have a high dose of steroids, because of the nerves… But we had to put the transplant back and back, because they couldn’t transplant me on such a high level of steroids. So we had to have a balance between the two conditions of getting the nerves to a point that they were under control, and that they wouldn’t relapse, but the steroid not being too high… so I was quite a unique case from that point of view, because renal and neuro had to really integrate with each [other] on a very regular basis.” (NCM04, man, 37, three LTC).

Such positive experiences of service integration could engender a sense of safety, where the needs of one condition were optimally balanced against another. However, there could be hidden difficulties integrating services in the hospital setting which create concerns for people living with MLTC, particularly regarding self-management. For example, one participant described difficulties self-managing his diabetes, leading to concerns about safe use of medication:

“You’re always waiting for a specialist to come and see you. You know? Like, somebody will come for my spleen, and then the nurse will say, ‘Oh, the fella for your spine is coming,’ but it’s quite late in the evening, really, sometimes. So the sheer number of people that deal with me, and my broken body, does make it hard, because you do a lot of waiting. And they might say, ‘Don’t have any lunch until you’ve seen…’ Dr, or Mr. …. But my food has a bearing on when I take my insulin, so then it does become important…because I could be putting more insulin in than my body can stand.” (NCM01, man, 72, five LTC).

b) Information systems

Participants reported difficulties with information transmission within the hospital setting including missing information and medication errors which required repeating their problems to multiple clinicians, occasionally receiving conflicting advice. Difficulties with information sharing could be exacerbated by receiving care for different conditions from two localities:

“So I’m split across the two boroughs… They work on different systems, so there’s no link. You know, there’s no… consistency with being able to see all your information, so they’ve got a bit on one system, they’ve got a bit on another” (NCM08, man, 62, three LTC).

While some reported that discharge summaries were comprehensive, others noted a range of problems including discovering unexplained medication changes, inaccurate information, or conditions they were unaware they had been diagnosed with, such as hospital-acquired pneumonia or non-alcoholic fatty liver disease. One woman noted that “all it said was about some tablets which I never received off them anyway” (NCF03, woman, 63, four LTC), while another participant had been given a summary for a procedure she had undergone the year before. Further, a participant reported a potentially serious information error:

“And when the letter came, and I got the copy for the GP, it had my name, and all my numbers on the top. It had the change of medication on the bottom, but it turned out to have somebody else’s full details in the middle” (NNM01, man, 79, three LTC).

c) Perceptions of clinical training and guidelines

Some participants perceived that clinicians were limited in their ability to deliver MLTC care due to their training and the clinical guidelines they were expected to use, which were described as “tick box” (NNF03, woman, 76, four LTC) and could constrain clinicians’ ability to respond flexibly to the needs of individuals in their decision-making processes. One participant noted the limitations of this approach, acknowledging that staff autonomy to make care decisions could be constrained by the organisational context:

“[Other conditions] are taken into account, but there’s a little bit of a flaw that they’re basically told to follow their certain pathway. Then, if A doesn’t work out, go to B. They follow it until it says no… So they’ll try to bend the rules but, at the end of the day, they’ve got masters to speak to as well so they can only do so much. So you’re listened to but there’s a way of doing it and that’s dictated to the staff.” (NCM10, man, 48, four LTC).

In addition, participants with rare conditions such as Still’s disease and uncommon tumours remarked on delayed diagnoses and a need for clinicians to have greater training and awareness of the conditions to improve care and to avoid potential interactions between medications. 

## Discussion

The findings from this study illuminate the experiences of people living with MLTC when they are admitted to hospital, represented in three themes spanning individual, interpersonal and organisational levels: internalised narratives of hospital care, alignment between clinician and patient knowledge, and organisational constraints on holistic care.

In recognition of the current limitations of siloed healthcare systems [31], much of the qualitative research evidence base on hospital care for people with more than one condition has highlighted the need for greater service integration and holistic person-centred care [32, 33]. By identifying some of the challenges experienced by people living with MLTC receiving inpatient care, the findings from this research echo that work, but also extend it by drawing attention to expectations of episodes of inpatient care, and how these are grounded in the context of hospital narratives of hospital care. Media reports or personal experiences of hospitals as an overwhelmed service, with long waits in Accident and Emergency departments and people receiving treatment on corridors [34], moderated expectations of care in the hospital setting, leading to expressions of surprise when efficient care was encountered.

Aside from medication provision, participants generally only expected treatment for the condition that had led to their admission. Inpatient hospital care was generally perceived as a discrete episode, an interruption in the daily flow of self-managing MLTC or seeking support from primary care services. Participants’ perceptions of hospital care were shaped by their experience of MLTC outside the hospital environment. In this regard, the biomedical definition of MLTC - living with more than one condition concurrently - may not fully align with lived experience and/or lived experience in the context of secondary care. As Porter et al. [35] note, the definition of MLTC is predicated on two basic assumptions, firstly that people conceptualise health and illness in relation to diagnostic labels and secondly that health conditions are phenomenologically experienced as co-existing. Porter et al. [35] argue that, rather than a lived experience of concurrence, LTC ‘slip in and out of apprehension according to their perception’ (p. 708). In this study, where the circumstances were such that many other conditions could be managed with medication, participants foregrounded the condition precipitating their admission. Hospital settings, generally understood as providing specialist short-term care for single conditions, may contribute to this foregrounding effect, enabling other conditions to become less visible in comparison to the presenting condition.

There were instances where self-management of LTC could be complicated by a tension between the routines of the hospital setting and the aim of participants to continue to enact their established practices [36, 37], whether through medication, resting when needed or use of incontinence products. Accustomed to self-management in their own homes, and with a tacit knowledge accrued through experience [37], people living with MLTC could feel anxious or frustrated when their medication regimens were not sustained in hospital, sometimes taking in their own medication or acquiring it through family members. Healthcare professionals should be aware of self-management practices to ensure that MLTC care is discussed within the hospital setting, incorporates patients’ experiential expertise into decision-making processes [25, 36] and is clinically safe, avoiding drug-drug interactions.

Although other LTC did not challenge the dominance of the condition leading to admission for all participants in this study, there were clearly circumstances of medical complexity when other conditions were or became clinically significant, and where integration between clinical specialties was needed. In these circumstances, cross-specialty communication was perceived as essential to optimise care delivery, and this study therefore resonates with other research that calls for integrated care models able to respond to the complexity of MLTC [38, 39].

The diversity of experience within our sample highlights the importance of avoiding the assumption that there is ‘one size fits all’ for people living with MLTC who are admitted to hospital. Services need to be flexible enough to respond to this heterogeneity, managing medically complex situations efficiently and acknowledging the experiential knowledge, self-management practices and complex prioritisation processes undertaken by people living with MLTC [40, 41]. Understanding the lived experience of MLTC, as beyond counts of conditions and as phenomenologically inconstant [33, 40, 41], will promote person-centred, individualised care approaches, the need for which has been highlighted in previous studies of hospital care for MLTC [31, 42].

There are striking parallels between the findings of this study and those of a qualitative study of frontline hospital clinicians’ approaches to decision-making for people with MLTC undertaken within the same research programme [26, 43]. Both studies have identified the tension between care for acute and chronic conditions within the predominantly specialist model of hospital care, resulting in uncertain pathways through the hospital system and inflexible care delivery or receipt. The fundamental misalignment between a system configured to provide single-organ care and the complex needs of people with MLTC entering hospital is exemplified by the constraining effects of single-condition clinical guidelines, which rarely offer explicit guidance on MLTC [44]. Referring to a set of clinical guidelines for people with MLTC can lead to conflicting care recommendations, riskier prescribing practices [45] and an increased treatment burden [46]. Embedding generalist skills through clinical training and professional development may be an important step in equipping hospital systems to provide care efficiently for the growing number of people with MLTC [16].

### Strengths and limitations

A key strength of this qualitative study is the exploration of experiences of a relatively large sample of people living with MLTC who had recently received inpatient hospital care. Further, having been collected in 2023 and 2024, the data are contemporaneous and relevant to hospital care in the post-COVID era. The patients in the sample are diverse in several respects, including age, gender, LTC and level of neighbourhood deprivation. Despite their heterogeneity, patterns of experience could be discerned which contribute to understanding the experience of MLTC in English hospitals.

Secondly, virtually all of the patients were admitted for unplanned care. Evidence indicates that people with MLTC have increased risk of emergency hospitalisation [47], with associated high costs [48]. Gaining insights into unplanned care is therefore important to address the challenge of reducing emergency admissions.

We acknowledge some limitations. We have less insight into experiences of people living with MLTC admitted to hospital for elective procedures or who stayed in hospital longer than a week. Secondly, while our sample was diverse in many respects, there was a lack of ethnic diversity due to the demographic profile of the areas served by the NHS recruiting sites. Concerted efforts to diversify the sample through other recruitment routes, including contacting voluntary sector agencies serving minority populations, were made but without success. We also acknowledge that people with intellectual disabilities were not represented in the sample, and only a small number of people with mental health conditions participated. Future research could address these limitations through engaging recruitment sites serving ethnically diverse populations, encouraging recruitment of people with MLTC which include intellectual disabilities and with mental health conditions, and recruiting people who had experienced planned admissions.

### Policy implications

This study has important policy implications for care provision. With high global prevalence of MLTC [8], healthcare systems worldwide are facing challenges sustaining services originally designed to treat single conditions. In England, for example, an independent investigation has recently described the NHS as being in ‘serious trouble’ [49] (p.1), identifying the inexorable rise in MLTC as a key factor intensifying the pressures on service delivery. In response, the 10 Year Health Plan for England [50] has set out radical transformations needed to create a sustainable health service capable of providing services to current and future populations, including a shift from hospitals to community settings. With this shift, the aim would be for care to be provided at home wherever possible, in neighbourhood health centres when further support is required, and only in hospital if absolutely necessary. Understanding the needs of people with MLTC currently accessing hospital care is an important step towards achieving this goal [7]. Implementing such radical changes in service delivery will need to be communicated effectively so that people living with MLTC are aware of how and where their care will be provided. Incorporating patient groups in planning the rollout of these transformations will be important to develop appropriate communication strategies. In addition, our data suggest that reconfigured services need to take account of the self-management practices and complex prioritisation processes of people living with MLTC in order to move closer towards delivery of person-centred care. We therefore propose that clinicians discuss self-management practices with people living with MLTC as soon as possible after admission, with the aim of identifying which established practices could be continued in the hospital setting or advising how self-management could be adapted during and after the inpatient stay.

## Conclusions

Healthcare systems that focus on the delivery of specialised care such as hospitals need transformation in order to address the increasing and complex challenge that MLTC presents. People living with MLTC who are admitted for inpatient hospital stays are highly diverse, with experience that goes beyond counts of conditions to incorporate fluctuating priorities, frustrations and concerns, and experience-based self-management practices. Redesigning hospital services to provide joined-up, person-centred care will require flexibility to respond to the wide spectrum of MLTC experiences. Involving people with lived experience of MLTC in the redesign of these processes is essential to ensure that hospital services can be reconfigured to adequately meet their needs.

## Supplementary Information

Below is the link to the electronic supplementary material.

Supplementary Material 1: Additional File 1 (.pdf): Interview topic guide employed with study participants

Supplementary Material 2: Additional File 2 (.pdf): Study participants’ health conditions and hospital stays: Number of self-reported health conditions, number of hospital stays in the last year and body systems affected by conditions (according to ICD-10 chapters)

Supplementary Material 3: Additional File 3 (.pdf): Additional sample demographics: marital status, household composition, current/ previous employment and educational attainment

Supplementary Material 4: Supplementary Information (.pdf): ADMISSION Research Collaborative Co-Investigators

## Data Availability

The data generated and analysed during the current study are not currently available due to ongoing follow-up data collection but will be archived in a university-managed dataset in future.

## Abbreviations

COPD: Chronic Obstructive Pulmonary Disease
ICD-10: The International Classification of Diseases, 10th revision
LTC: Long-term Conditions
MLTC: Multiple Long-term Conditions
NHS: National Health Service
NIHR: National Institute for Health and Care Research
PAG: Patient Advisory Group
UK: United Kingdom
