# Supplementary material for: ‘People need to be a bit more understanding that my body is wrecked’: A qualitative exploration of inpatient hospital care for people living with multiple long-term conditions
Source: BMC Med. 2026 Oct 16;24:567. doi: 10.1186/s12916-026-05181-4 (PMC13673762; doi:10.1186/s12916-026-05181-4)
Supplement: Supplementary file 2 [file 12916_2026_5181_MOESM2_ESM.docx]

**Additional file 2 : Number of self-reported health conditions, number of hospital stays in the last year and body systems affected by conditions (according to ICD-10 chapters)**

| **Partici-pant**  **ID code** | **Age** | **No. of self-reported conditions** | **No. of hospital stays in last year** | **Cancer** | **Cardio-vascular disease** | **Digestive**  **Disease** | **Ear**  **disease** | **Eye**  **disease** | **Haema-tological**  **disorder** | **Infectious**  **disease** | **Mental / behaviour-al disorder** | **Metabolic/**  **endocrine**  **disorder** | **Musculo-skeletal**  **disease** | **Neuro-**  **logical**  **disease** | **Respir-atory disease** | **Urogenital disorder** |
| --- | --- | --- | --- | --- | --- | --- | --- | --- | --- | --- | --- | --- | --- | --- | --- | --- |
| GHM01 | 69 | 11 | >=5 |  |  |  |  |  |  |  |  |  |  |  |  |  |
| GHM02 | 75 | 3 | >=5 |  |  |  |  |  |  |  |  |  |  |  |  |  |
| NCF01 | 83 | 6 | 2-4 |  |  |  |  |  |  |  |  |  |  |  |  |  |
| NCF02 | 72 | 4 | 1 |  |  |  |  |  |  |  |  |  |  |  |  |  |
| NCF03 | 63 | 4 | 1 |  |  |  |  |  |  |  |  |  |  |  |  |  |
| NCF04 | 71 | 6 | 2-4 |  |  |  |  |  |  |  |  |  |  |  |  |  |
| NCF05 | 76 | 2 | 1 |  |  |  |  |  |  |  |  |  |  |  |  |  |
| NCF06 | 75 | 6 | 1 |  |  |  |  |  |  |  |  |  |  |  |  |  |
| NCF07 | 86 | 5 | 1 |  |  |  |  |  |  |  |  |  |  |  |  |  |
| NCF08 | 77 | 4 | 1 |  |  |  |  |  |  |  |  |  |  |  |  |  |
| NCF09 | 62 | 3 | 1 |  |  |  |  |  |  |  |  |  |  |  |  |  |
| NCF10 | 60 | 6 | 1 |  |  |  |  |  |  |  |  |  |  |  |  |  |
| NCM01 | 72 | 5 | 2-4 |  |  |  |  |  |  |  |  |  |  |  |  |  |
| NCM02 | 78 | 6 | 2-4 |  |  |  |  |  |  |  |  |  |  |  |  |  |
| NCM03 | 80 | 4 | 1 |  |  |  |  |  |  |  |  |  |  |  |  |  |
| NCM04 | 37 | 3 | 1 |  |  |  |  |  |  |  |  |  |  |  |  |  |
| NCM05 | 81 | 4 | 2-4 |  |  |  |  |  |  |  |  |  |  |  |  |  |
| NCM06 | 71 | 4 | 2-4 |  |  |  |  |  |  |  |  |  |  |  |  |  |
| NCM07 | 84 | 3 | 2-4 |  |  |  |  |  |  |  |  |  |  |  |  |  |
| NCM08 | 62 | 3 | 1 |  |  |  |  |  |  |  |  |  |  |  |  |  |
| NCM09 | 74 | 3 | 1 |  |  |  |  |  |  |  |  |  |  |  |  |  |
| NCM10 | 48 | 4 | 1 |  |  |  |  |  |  |  |  |  |  |  |  |  |
| NNF01 | 64 | 2 | 2-4 |  |  |  |  |  |  |  |  |  |  |  |  |  |
| NNF02 | 39 | 3 | 2-4 |  |  |  |  |  |  |  |  |  |  |  |  |  |
| NNF03 | 76 | 4 | 1 |  |  |  |  |  |  |  |  |  |  |  |  |  |
| NNF04 | 75 | 6 | 1 |  |  |  |  |  |  |  |  |  |  |  |  |  |
| NNM01 | 79 | 3 | 2-4 |  |  |  |  |  |  |  |  |  |  |  |  |  |
| NNM02 | 72 | 5 | 2-4 |  |  |  |  |  |  |  |  |  |  |  |  |  |
| NTF01 | 77 | 6 | 1 |  |  |  |  |  |  |  |  |  |  |  |  |  |
| NTF02 | 85 | 6 | 2-4 |  |  |  |  |  |  |  |  |  |  |  |  |  |
| NTF03 | 80 | 5 | 1 |  |  |  |  |  |  |  |  |  |  |  |  |  |
| NTF04 | 68 | 5 | 2-4 |  |  |  |  |  |  |  |  |  |  |  |  |  |
| NTF05 | 34 | 3 | 1 |  |  |  |  |  |  |  |  |  |  |  |  |  |
| NTF06 | 40 | 4 | >=5 |  |  |  |  |  |  |  |  |  |  |  |  |  |
| NTF07 | 58 | 4 | 2-4 |  |  |  |  |  |  |  |  |  |  |  |  |  |
| NTF08 | 50 | 2 | 2-4 |  |  |  |  |  |  |  |  |  |  |  |  |  |
| NTF09 | 54 | 5 | 1 |  |  |  |  |  |  |  |  |  |  |  |  |  |
| NTM01 | 82 | 6 | >=5 |  |  |  |  |  |  |  |  |  |  |  |  |  |
| NTM02 | 75 | 6 | 2-4 |  |  |  |  |  |  |  |  |  |  |  |  |  |
| NTM03 | 72 | 4 | 2-4 |  |  |  |  |  |  |  |  |  |  |  |  |  |
| NTM04 | 87 | 3 | 1 |  |  |  |  |  |  |  |  |  |  |  |  |  |
| NTM05 | 72 | 3 | 1 |  |  |  |  |  |  |  |  |  |  |  |  |  |
| NTM06 | 65 | 4 | 1 |  |  |  |  |  |  |  |  |  |  |  |  |  |
| NTM07 | 51 | 6 | >=5 |  |  |  |  |  |  |  |  |  |  |  |  |  |
