# Supplementary material for: ‘People need to be a bit more understanding that my body is wrecked’: A qualitative exploration of inpatient hospital care for people living with multiple long-term conditions
Source: BMC Med. 2026 Oct 16;24:567. doi: 10.1186/s12916-026-05181-4 (PMC13673762; doi:10.1186/s12916-026-05181-4)
Supplement: Supplementary file 3 [file 12916_2026_5181_MOESM3_ESM.docx]

**Supplementary Information: ADMISSION Research Collaborative Co-investigators**

Victoria Bartle^1^

Rachel Cooper^2,3^

Heather J Cordell^4^

Ray Holding^1^

Tom P Marshall^5^

Fiona E Matthews^6^

Paolo Missier^7^

Ewan R Pearson^8^

Chris Plummer^3,9^

Sian M Robinson^2,3^

Elizabeth Sapey^10, 11^

Avan A Sayer^2,3^

Thomas Scharf^4^

Mervyn Singer^12,13^

James MS Wason^14^

Miles D Witham^2,3^

1. Public Co-Investigator, ADMISSION Research Collaborative, Newcastle upon Tyne, UK
2. AGE Research Group, Translational and Clinical Research Institute, Faculty of Medical Sciences, Newcastle University, Newcastle upon Tyne, UK
3. NIHR Biomedical Research Centre: Newcastle, Newcastle upon Tyne Hospitals NHS Foundation Trust, Cumbria, Northumberland, Tyne and Wear NHS Foundation Trust and Newcastle University, Newcastle upon Tyne, UK
4. Population Health Sciences Institute, Faculty of Medical Sciences, Newcastle University, Newcastle upon Tyne, UK
5. Institute of Applied Health Research, University of Birmingham, Birmingham, UK
6. Research and Enterprise Office, University of Hull, Hull, UK
7. School of Computer Science, University of Birmingham, Birmingham, UK
8. Division of Population Health and Genomics, Ninewells Hospital and School of Medicine, University of Dundee, Dundee, UK
9. Digital Services, Newcastle upon Tyne Hospitals NHS Foundation Trust, Newcastle upon Tyne, UK
10. PIONEER Hub, University of Birmingham, Birmingham, UK.
11. Institute of Inflammation and Ageing, University of Birmingham, Birmingham, UK.
12. University College London Hospitals NHS Foundation Trust, London, UK
13. Bloomsbury Institute for Intensive Care Medicine, University College London, London, UK
14. Biostatistics Research Group, Population Health Sciences Institute, Newcastle University, Newcastle upon Tyne, UK
