# Supplementary material for: ‘People need to be a bit more understanding that my body is wrecked’: A qualitative exploration of inpatient hospital care for people living with multiple long-term conditions
Source: BMC Med. 2026 Oct 16;24:567. doi: 10.1186/s12916-026-05181-4 (PMC13673762; doi:10.1186/s12916-026-05181-4)
Supplement: Supplementary file 4 [file 12916_2026_5181_MOESM4_ESM.docx]

**Additional file 3: Additional demographic characteristics of study participants**

| **Characteristic** | **Frequency**  **N (%)** |
| --- | --- |
| **Marital status** |  |
| Married or co-habiting | 25 (57) |
| Widowed | 9 (20) |
| Divorced | 6 (14) |
| Single | 4 (9) |
| **Household composition** |  |
| Lives with spouse/ partner | 18 (45) |
| Lives with spouse and adult child/children | 5 (11) |
| Lives with spouse and young child/ children | 2 (5) |
| Lives alone | 16 (36) |
| Lives with adult child/children | 2 (5) |
| Lives with young child/ children | 1 (2) |
| **Current / previous employment** |  |
| Senior professional / manager (e.g. architect, lawyer, director)   1. Retired 2. Employed | 7 (16)  1 (2) |
| Skilled professional (e.g. nurse, police officer, teacher, electrician)   1. Retired 2. Employed | 10 (23)  2 (5) |
| Manual worker (e.g. driver, coal miner)   1. Retired 2. Employed | 11 (25)  0 (0) |
| Administrator   1. Retired 2. Employed | 10 (23)  1 (2) |
| Retired (other) | 1 (2) |
| Unemployed | 1 (2) |
| **Educational attainment** |  |
| Left school before age 16 | 8 (18) |
| GCSEs or equivalent | 8 (18) |
| A levels | 2 (5) |
| Further education qualification | 13 (30) |
| Undergraduate degree or equivalent | 11 (25) |
| Postgraduate degree (Master’s) | 2 (5) |
